# *In Vitro* Inhibition of *Helicobacter pylori* Growth by Redox Cycling Phenylaminojuglones

**DOI:** 10.1155/2018/1618051

**Published:** 2018-04-24

**Authors:** Julio Benites, Héctor Toledo, Felipe Salas, Angélica Guerrero, David Rios, Jaime A. Valderrama, Pedro Buc Calderon

**Affiliations:** ^1^Facultad de Ciencias de la Salud, Universidad Arturo Prat, Casilla 121, 1100000 Iquique, Chile; ^2^Instituto de Ciencias Exactas y Naturales, Universidad Arturo Prat, Casilla 121, 1100000 Iquique, Chile; ^3^Instituto de Ciencias Biomédicas (ICBM), Facultad de Medicina, Universidad de Chile, 8380453 Santiago, Chile; ^4^Research Group in Metabolism and Nutrition, Louvain Drug Research Institute, Université catholique de Louvain, Louvain-la-Neuve, Belgium

## Abstract

Infection by *Helicobacter pylori* increases 10 times the risk of developing gastric cancer. Juglone, a natural occurring 1,4-naphthoquinone, prevents *H. pylori* growth by interfering with some of its critical metabolic pathways. Here, we report the design, synthesis, and *in vitro* evaluation of a series of juglone derivatives, namely, 2/3-phenylaminojuglones, as potential *H. pylori* growth inhibitors. Results show that 5 out of 12 phenylaminojuglones (at 1.5 *μ*g/mL) were 1.5–2.2-fold more active than juglone. Interestingly, most of the phenylaminojuglones (10 out of 12) were 1.1–2.8 fold more active than metronidazole, a known *H. pylori* growth inhibitor. The most active compound, namely, 2-((3,4,5-trimethoxyphenyl)amino)-5-hydroxynaphthalene-1,4-dione 7, showed significant higher halo of growth inhibitions (HGI = 32.25 mm) to that of juglone and metronidazole (HGI = 14.50 and 11.67 mm). Structural activity relationships of the series suggest that the nature and location of the nitrogen substituents in the juglone scaffold, likely due in part to their redox potential, may influence the antibacterial activity of the series.

## 1. Introduction

*Helicobacter pylori* (*H. pylori*) is a Gram-negative bacillary spiral-shaped bacterium that colonizes the human stomach [1] and is associated with a number of human diseases, including gastritis, peptic ulceration, and gastric cancer [2, 3]. Due to its direct incidence in human cancer, *H. pylori* belongs to the group 1 of carcinogens according to the International Agency for Research on Cancer (IARC) [4, 5]. In the stomach, as part of its mechanism of survival adaptation, *H. pylori* express high levels of urease, converting urea into ammonium and carbonic anhydride. This creates an alkaline local medium that allows the survival of *H. pylori* in the acidic environment of the stomach, facilitating the colonization of the gastric mucosa [6–8]. Frequently, *H. pylori* infection is acquired during childhood, and if it is not treated, it may remain throughout the entire patient life [9]. Approximately 50% of the world population is chronically infected with *H. pylori* [1, 10–12] but most of the patients are asymptomatic [13, 14]. In spite of the fact that only a fraction of the infected population develops a severe pathology, it has been estimated that the risk of developing gastric cancer is increased 10 times upon *H. pylori* infection [5].

Currently, the eradication treatment of *H. pylori* includes a double antibiotic therapy plus a proton pump inhibitor. This high-cost treatment regimen is often problematic (failure rates between 20 and 40%), with undesirable side effects that limit patient compliance and lead to the selection of antibiotic-resistant bacteria [15–17]. Lower incidence of infection with *H. pylori* has been associated with the consumption of many food of vegetal origin, including wine and green tea which are rich in phytochemicals such as flavones, isoflavones, flavo- and flavanols, anthocyanidins, tannins, and stilbene derivatives [18–21]. Taken together, it is necessary to find new therapies that would help to eradicate *H. pylori* infection and prevent gastric cancer [22].

Quinones represent an important class of naturally occurring compounds that are widely found in animals, plants, and microorganisms [23]. These compounds act as inhibitors of electron transport and uncouplers of oxidative phosphorylation and give rise to a wide range of cytostatic and antiproliferative activities [24]. 1,4-Naphthoquinones (i.e., juglone, plumbagin, and lawsone) are an interesting subgroup of quinones that displays remarkable biological properties [25–27]. The biological activity shown by 1,4-naphthoquinones relies upon their ability to accept one and/or two electrons to form radical anion or hydroquinone [28], which leads to the generation of reactive oxygen species (ROS) such as hydrogen peroxide and superoxide that cause cell damage [29]. It has been reported that hydrophobic and steric factors may be determinants on the biological activity in 1,4-naphthoquinones [30].

Phytochemicals that display antimicrobial activity may inhibit *H. pylori* growth by different mechanisms to those reported for standard antibiotic drugs and could be used as an alternative approach to avoid the development of bacterial resistance. Regarding the antimicrobial effects mediated by quinones, they act on cell surface-exposed molecules, cell wall polypeptides, and membrane-bound enzymes of *H. pylori*. For instance, juglone is a promising inhibitor of *H. pylori* growth because of its capacity to interfere with essential processes such as inhibition of 3 key *H. pylori* enzyme activities: cystathionine *γ*-synthase (HpCGS), malonyl-CoA acyl carrier protein transacylase (HpFabD), and *β*-hydroxyacyl-ACPdehydratase (HpFabZ) [31]. The anti-*H. pylori* activity of several quinones, including juglone, menadione, and plumbagin, has been shown by MIC_90_ values around 0.8–25 *μ*g/mL [18]. Meanwhile, lawsone analogs have shown inhibitory activity against the membrane-embedded protein quinol/fumarate reductase (QFR) from *Wolinella succinogenes*, a target closely related to QFRs from *H. pylori* [32]. Moreover, other 1,4-napthoquinone derivatives, such as 2-methoxy-1,4-naphthoquinone, also display a strong anti-*H. pylori* activity [18, 33]. Finally, a series of 2-hydroxy-1,4-naphthoquinones showed activity against *H. pylori* by acting on bacterial thymidylate synthase [34].

The aim of the study was to design new anti-*H. pylori* agents. To this end, a series of 2- and 3-phenylaminojuglone-based substances was prepared from juglone to assess their anti-*H. pylori* activity. In addition, we evaluated the influence of stereoelectronic and hydrophobic parameters of these compounds on the anti-*H. pylori* activity.

## 2. Materials and Methods

### 2.1. Preparation of Phenylaminojuglone Derivatives: General Procedure

**Scheme 1:**
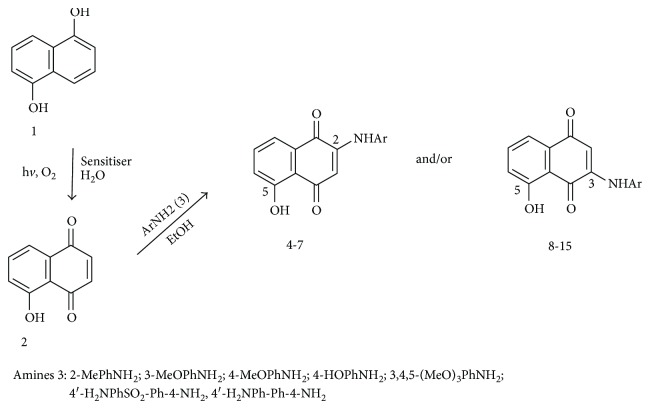
Preparation of the 2/3-phenylaminojuglones 4–15.

**Figure 1:**
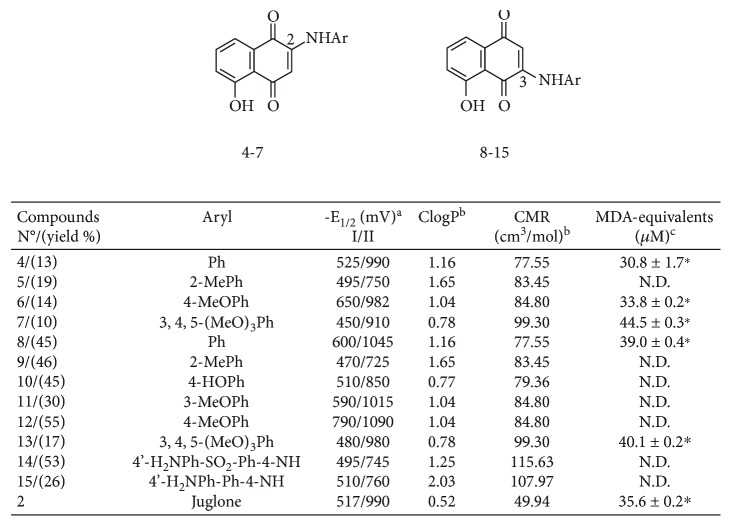
Yields, physicochemical descriptors, and MDA equivalent values of compounds **4**–**15**. ^a^*E*_1/2_ = first and second halfwave potential. ^b^Determined by the ChemBioDraw Ultra 11.0 software. ^c^The formation of MDA equivalents was performed as reported in Material and Methods. Selected quinone compounds were tested at 1 mM. Values obtained by quinones were compared to MDA produced under control conditions (iron salts plus deoxyribose) which was 21.0 ± 0.5 MDA equivalents (*μ*M). ^∗^*p* < 0.05 as compared to control conditions values. N.D.: not determined.

Suspensions of 1,5-dihydroxynaphthalene (1; 1.25 mmol), rose bengal (20 mg; 0.02 mmol), and water (150 mL) were exposed to green LEDs for 5 h while a gentle stream of air was bubbled through the solution. Thereafter, phenylamines 3 (1.5 mmol) were added and the solutions were stirred for 4 h at room temperature (RT). Work-up of the reaction mixtures followed by column chromatography over silica gel (3 : 1 petroleum ether/ethyl acetate) provided pure compounds 4–15 (Scheme 1; Figure 1).

All reagents were of commercial quality and used without further purification. The melting points were measured in a Stuart Scientific SMP3 equipment. The IR spectra were obtained in a vector 22-FT Bruker spectrophotometer using KBr disks, and wavelengths are expressed in cm^−1^. Proton nuclear magnetic resonance (^1^H NMR) spectra were measured at 400 and 300 MHz in a Bruker AM-400 and Ultrashield-300 spectrometers. Chemical shifts are expressed in ppm using TMS as an internal reference (*δ* scale), and (*J*) coupling constants are expressed in hertz (Hz). Carbon-13 nuclear magnetic resonance (^13^C NMR) spectra were measured at 100 and 75 MHz in a Bruker AM-400 and Ultrashield-300, spectrometers. Silica gel (70–230 and 230–400 mesh) and TLC on aluminum foil 60 F_254_-supported silica (Merck, Darmstadt) were used for the chromatography analytical columns and TLC, respectively.

### 2.2. Calculation of Molecular Descriptors

Calculation of lipophilicity (ClogP) and molar refractivity (CMR) was assessed by using the ChemBioDraw Ultra 11.0 software and the obtained values are shown in Figure 1. Redox potentials of juglone and phenylaminojuglones were measured by cyclic voltammetry at room temperature (RT) in acetonitrile as solvent using a platinum electrode and 0.1 M tetraethylammonium tetrafluoroborate as the supporting electrolyte [35]. It should be noted that in aqueous solution by using pulse radiolysis, a different redox potential value of juglone is obtained [36, 37]. Well-defined quasi-reversible waves, the cathodic peak related to the reduction of quinone, and the anodic one due to its reoxidation, were observed for the compounds. The voltammograms were run in the potential range from 0 to −2.0 V versus nonaqueous Ag/Ag^+^. The first and the second halfwave potential values (E^I^_1/2_) of juglone and phenylaminojuglones, evaluated from the voltammograms obtained at a sweep rate of 100 mV s^−1^, are summarized in Figure 1.

### 2.3. Biological Activity

#### 2.3.1. Reagents

Cellulose acetate filters, sodium chloride, and bacto agar were purchased at Asahi Glass, (Tokyo, Japan) and JT Baker (Mexico), respectively. Metronidazole was from Sigma Aldrich (St. Louis, MO 63103, USA). All other chemicals were ACS reagent grade. Stock solutions of juglone and its analogs were prepared by dissolving 50 mg of the compound in 1 mL of 100% DMSO. Solutions were sterilized by filtration through cellulose acetate filters (0.2 mm pore size; 25 mm diameter).

#### 2.3.2. Bacterial Strain and Growth Conditions

*H. pylori* 26695 (ATCC 700392), isolated from a United Kingdom patient with gastritis, was obtained from the American Type Culture Collection (Manassas, VA, USA). Frozen stocks of *H. pylori* were recovered and routinely grown for 48 h at 37°C, 5.5% CO_2_, and 70 to 80% relative humidity on Trypticase soy agar plates (TSA) from Becton Dickinson (Sparks, MD 21152, USA) supplemented with 0.4% *H. pylori* selective supplement Dent (Oxoid Basingstoke, Hampshire, England), 0.3% IsoVitalex (Oxoid), and 5% horse serum from Thermo Fisher Scientific HyClone (Utah 84321, USA) [38, 39]. For liquid growth experiments, cells were grown in Trypticase soy broth (TSB) (Becton Dickinson) with 5% horse serum, supplemented with IsoVitalex and Dent (Oxoid). Bacteria were first grown to an optical density of 0.6 to 1.0 at 600 nm (OD_600_) at pH 7.0 and subsequently diluted to a starting OD_600_ of 0.05. To measure the growth of *H. pylori* in liquid medium, a serial dilution was prepared, aliquots of the various dilutions were plated on Trypticase soy agar plates, and the number colony-forming units (CFU) was determined [40].

#### 2.3.3. *H. pylori* Growth Assay in Liquid Medium

*H. pylori* (3 × 10^7^ cells/mL) were inoculated in 5 mL of TSB and supplemented with a range of concentrations (0.0 to 1.0 *μ*g/mL) of juglone or a derivative compound. After incubation at 37°C for 48 h with constant shaking at 250 rpm in a controlled atmosphere (5.5% CO_2_ and 70% relative humidity), bacterial growth was determined by turbidimetry at 600 nm or by counting colony-forming units on TSA plates [41, 42].

#### 2.3.4. *H. pylori* Viability Assay

From each of the experimental culture tubes described in the previous section, 100 *μ*L aliquots were taken at the end of the incubation period to prepare serial dilutions in PBS. Aliquots of 10 *μ*L from each of these dilutions were plated on TSA and incubated for 48 h at 37°C [43]. The number of colony-forming units per mL (CFU/mL) corresponding to each experimental condition was determined.

#### 2.3.5. Inhibition Halo Test on Agar Plates

The procedure was performed as described by Rodríguez et al. [44]. One hundred *μ*L of *H. pylori* suspension containing 3 × 10^7^ cells/mL was evenly spread over the TSA plates with a metal handle loop. Then, three-millimeter diameter wells were made in the plates and 30 *μ*L of a series of compound solutions was deposited in the wells (corresponding to 0 to 1 mg/well). After 48 h of incubation at 37°C, the diameter of the growth inhibition halos was determined.

#### 2.3.6. Determination of Prooxidant Activity

The assay was based on TBARS method according to Halliwell et al. [45]. Briefly, a mixture containing iron salts, phosphate buffer, and deoxyribose was incubated for 60 min at RT in the absence or presence of quinones. Then, the amount of malondialdehyde (MDA) equivalent produced was determined by reaction with thiobarbituric acid and further reading at 532 nm. Results are expressed as *μ*M of MDA equivalents. The prooxidant activity of some selected quinones is shown in Figure 1.

#### 2.3.7. Statistical Analysis

All experiments were performed at least 3 times and groups were compared by ANOVA test using GraphPad Prism software (San Diego, CA 92037, USA). Two-way ANOVA test was used to analyze the dose-response curves. A *p* value < 0.05 was set as statistically significant.

## 3. Results

### 3.1. Synthesis of Phenylaminojuglones

The preparation of the phenylaminojuglone derivatives was achieved via a two synthetic step sequence from 1,5-dihydroxynaphthalene 1 and the selected phenylamines 3 according to 1 and Figure 1. In the first step, sensitized photooxygenation of compound 1 on water gave 5-hydroxy-1,4-naphthoquinone (2, juglone) in 64% yield [46]. Further reaction of juglone 2 with the phenylamines in ethanol [47, 48] at room temperature provided the respective phenylaminojuglones 4–15. In all cases, the reaction gave a mixture of the respective regioisomers as was observed by thin layer chromatography and proton magnetic resonance. Pure samples of the regioisomers 4–7 (C-2) and 8–15 (C-3) were isolated by column chromatography (Figure 1). Efforts to isolate minor regioisomers were unsuccessful. The formation of regioisomers in these reactions reveals that they proceed under regiochemical control. The structures of the phenylaminojuglones were established by nuclear magnetic resonance (^1^H-NMR and ^13^C-NMR) and high-resolution mass spectrometry (HRMS). The location of the phenylamino substituents at the quinone nucleus in compounds 4–7 and 8–15 was determined by bidimensional nuclear magnetic resonance (2D-NMR) (data in the Supplementary Material available here).

### 3.2. Inhibition of *H. pylori* Growth by Juglone, Phenylaminojuglones, and Metronidazole

To assess the effect of juglone and its analogs on *H. pylori* growth, increasing doses of compounds were added into TSA well-plates previously seeded with bacteria, which were further incubated for 48 h.  Table 1 shows the halo of growth inhibition (HGI) in millimeter obtained for each compound as a function of their concentration by using the Diffusion Test assay.

Juglone and most of its analogs (except 5 and 9) were more active on *H. pylori* than metronidazole (HGI: 11.67 mm). Compared to the antibacterial effect mediated by juglone (HGI: 14.50 mm), 5 out of 12 phenylaminojuglones were more efficient than juglone with HGI values ranging from 22.25 to 32.25 mm. A clear representation of this inhibitory effect is unveiled when the antimicrobial activity of compounds based on molar amounts was compared. For instance, the HGI of 33.25 mm of 7 was obtained at 4.5 *μ*M while the HGI of juglone (14.50 mm) and metronidazole (11.67 mm) were obtained at 9.2 and 9.35 *μ*M, respectively. In other words, 7 reached a high inhibitory effect on *H. pylori* growth at half of the doses required by juglone and metronidazole whose effects were by far lower than **7**.

The C-H functionalization in the 1,4-naphthoquinone scaffold at either C-2 or C-3, like in the pairs 4/8, 5/9, 6/12, and 7/13, resulted in similar antibacterial activities as shown by their halo of inhibition. For instance, 4 and 8 have an HGI of 13.25 and 13.75 mm, respectively, and 7 and 13 have an HGI of 32.25 and 28.50 mm, respectively.

Compound 14, obtained by oxidative amination of 2 with dapsone (4-H_2_NPhSO_2_Ph-4′-NH_2_), displayed higher inhibitory activity on *H. pylori* growth than juglone at all the tested doses. Since dapsone may act against bacteria by inhibiting the synthesis of dihydrofolic acid [49] it is likely that such antimicrobial ability mediated by dapsone is contributing to the overall anti-*pylori* activity of 14. Finally, arylaminojuglone 15 derived from 2 and benzidine (4-H_2_NPh-Ph-4′-NH_2_) showed a lower range of activity than juglone. It should be noted that amines 3 phenylamine, 2-methylphenylamine, 3-methoxyphenylamine, 4-methoxyphenylamine, 4-hydroxyphenylamine, 3,4,5-trimethoxyphenylamine, and benzidine were devoid of anti-*pylori* activity when added in the absence of juglone (data not shown).

### 3.3. *H. pylori* Viability in the Presence of Juglone, 7, and Metronidazole

Once determining the effect of quinone-derived compounds in solid medium (halo of growth inhibition assay), we investigated the effect of juglone and the more active phenylaminojuglone (**7**) on *H. pylori* viability in liquid medium. To this end, TSB medium was supplemented with increasing concentrations of each compound and incubated for 48 h. Next, aliquots were removed and colony- forming units (CFU) were counted. Bacteria viability results are expressed as CFU/mL.

**Figure 2:**
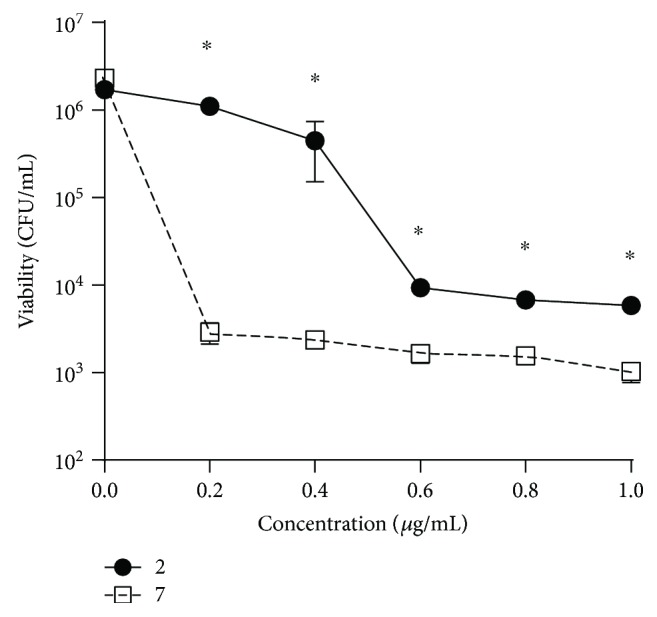
Effect of Juglone 2 and 7 on *H. pylori* viability in liquid medium. (^∗^) Statistically significant differences (*p* < 0.05) between 2 and 7.

Figure 2 shows a dose-dependent decrease of CFU/mL values from 1.71 × 10^6^ to 5.85 × 10^3^ when *H. pylori* was incubated with juglone. Likewise, CFU/mL values decreased from 2.3 × 10^6^ to 1.04 × 10^3^ CFU/mL when *H. pylori* was incubated with compound 7. Interestingly, although bacteria viability is significantly decreased in a dose-dependent manner in both conditions, some marked differences were noted: First, at low doses (0.2 *μ*g/mL), compound 7 reduced dramatically the viability of *H. pylori* while juglone, at the same concentration, did not affect significantly the bacteria viability. Second, it is required to use 0.6 *μ*g/mL of juglone in order to reach the inhibitory effect of 7 (0.2 *μ*g/mL) on the growth of *H. pylori* (3-fold increase). In terms of molarity, as we have previously shown, such difference is even higher in favor to 7. Indeed, 0.2 *μ*g/mL of 7 corresponds to 0.56 *μ*M, while 0.6 *μ*g/mL of juglone corresponds to 3.44 *μ*M, a difference of 6-fold to obtain similar inhibitory effects.

**Figure 3:**
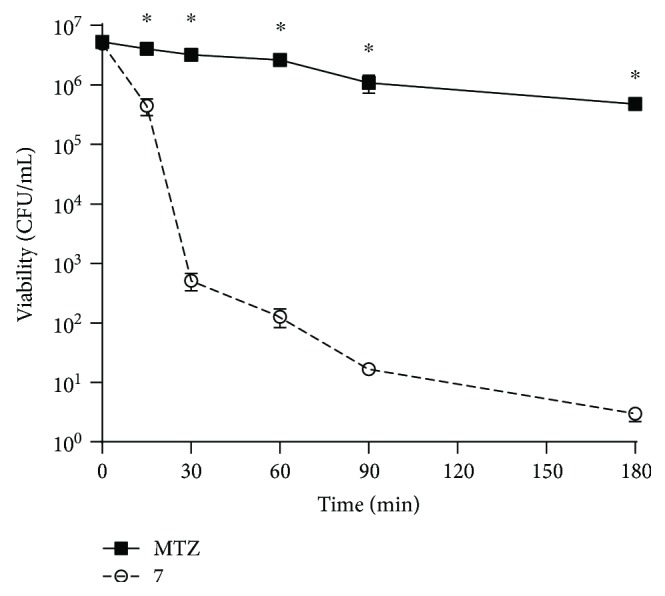
Kinetic course of *H. pylori* 26695 growth inhibition in the presence of 7 and metronidazole, both at 0.8 *μ*g/mL, during 180 min. ^∗^*p* < 0.05 between metronidazole and 7.

Figure 3 shows the bacteria viability during 180 min of incubation in the presence of metronidazole and 7 both at doses of 0.8 *μ*g/mL. Compound 7 provoked a rapid and strong inhibition of *H. pylori* growth decreasing the CFU values from 4.95 × 10^6^ to 5.15 × 10^2^ 30 minutes after incubation. In contrast, metronidazole slightly decreased the CFU values from 5.3 × 10^6^ at the beginning of the incubation to 3.23 × 10^6^ after 30 min. At the end of the 180 min of incubation, the CFU value for metronidazole was still high reaching 4.80 × 10^5^ whereas 7 practically causes a total loss of the bacteria. It should be noted that in terms of molarity, 7 was tested at 2.25 *μ*M while metronidazole was used at 4.7 *μ*M, highlighting the ability of 7 as a potential anti-*pylori* molecule.

## 4. Discussion

Traditional medicine used by ancient cultures relies on the use of natural compounds with biological activity, which can be used as starting molecules to modify their structures for improving their pharmacological properties. The aim of this work was to synthesize a series of phenylaminojuglones with anti-*H. pylori* biological activity. Among the members of the series, five congeners were found 1.9- to 2.8-times more active than one standard therapeutic drug (i.e., metronidazole), a currently standard anti-*pylori* drug [40, 50–52].

Even though the discovery of molecular mechanisms underlying the antibacterial effects of the phenylaminojuglones was beyond our objectives, we noted that their anti-*pylori* activity depends on the nature and location of the nitrogen substituents at the quinone nucleus of the juglone scaffold. Thus, insertion of the 3,4,5-trimethoxyphenylamino group at the 2 position in juglone, as in compound 7 (HGI: 32.25 mm), induced a strong effect on the antibacterial activity of the juglone scaffold (HGI: 14.50 mm). Conversely, the insertion of the phenylamino, 2-phenylamino, and 4-methoxyphenylamino groups, as in compounds 4 (HGI: 13.25 mm), 5 (HGI: 11.00 mm) and 6 (13.50 mm), causes decreasing effects on the antibacterial activity of the juglone scaffold. Inspection of Table 1 reveals that, in general, the insertion of the nitrogen substituents in the 3 position induce higher effects on the antibacterial activity compared to the insertion of nitrogen substituents in the 2 position of the juglone scaffold. Among the members of the 3-arylaminojuglone derivatives 8–15, compounds 10, 12, and 13 display remarkable antibacterial activities. Once again, in terms of molarity, such high doses (1.6 *μ*g/mL) correspond to 6 *μ*M of compound 4, 4.5 *μ*M of compound 7, and 9.2 *μ*M of juglone, strengthening the assumption about the efficacy of compound 7.

By comparing data from Figure 1 and Table 1 (HGI of juglone and its analogs as well as molecular descriptors), it can be inferred that compounds 7, 10, and 13 (three of the most active phenylaminojuglones) have lower ClogP values than other molecules of the series (around 0.78), showing a marked hydrophilic character. Moreover, when compared with compounds 9 and 13 that share similar values of redox potential and polarizability but different lipophilia, they have strong differences in terms of anti-*pylori* activity: HGI: 10.75 and 28.50 mm, respectively. It appears then that compounds with a significant hydrophilic degree will have a more pronounced antibacterial activity. Interestingly, it has been reported that the membrane surface of *H. pylori* is rather hydrophilic and it is negatively charged [53]. This property would facilitate the entry of these molecules inside the bacteria, facilitating their biological activity. This tempting hypothesis is however unlike because juglone has a ClogP value (0.52) even lower than the three former molecules but its HGI was only of 14.5 mm.

It is to be expected that the redox status of the cellular system would be modulated by ROS. Since the ease of ROS generation through reduction of a quinonoid would depend on its electrochemical parameters, the redox potential of a quinone would influence its overall biological profile, which encompasses the functional, toxicological, mutagenic, and antitumor activities. With this view, the redox potential of these naphthoquinones was determined by cyclic voltammetry using acetonitrile, an aprotic solvent, which mimics the environment of the cell membrane [54]. Figure 1 shows that *E*_1/2_ values for the first one electron transfer, corresponding to the formation of the radical-anions of compounds 4–15, are spread into a broad potential range from −790 to −450 mV. In addition, we noted that 7 out of 12 phenylaminojuglones have higher redox potential values (from −450 to −510 mV) than juglone (−517 mV). It is tempting to assume that the effect on redox potential by the insertion of nitrogen groups, such as PhNH-, in the 5-hydroxy-1,4-naphthoquinone (juglone) scaffold may be clearly predicted, but the situation is a little bit more complex. Indeed, it is reasonable to assume that the electron acceptor ability of these phenylaminojuglone will depend, in part, on the location of the nitrogen donor in the quinone core and on the extent of the conjugative effect of this group to the intramolecular hydrogen bond of the molecule. A similar situation by taking the 1,4-naphthoquinone scaffold has been discussed by Aguilar-Martinez et al. [55].

Regarding the influence of other molecular descriptors such as molar refractivity, it seems that high polarizability values enhance the anti-*pylori* activity. Indeed, when compared compounds 5 and 14, they have the same redox potential values (−495 mV) and similar lipophilia (1.65 versus 1.25) but different polarizability values. Accordingly, compound 14 with a high molar refractivity (115.63), it has a high anti-*pylori* activity (HGI: 22.25 mm). However, compound 7 has less lipophilicity, high polarizability and low redox potential compared to 12, and their HGI values were markedly different: 32.25 and 12.75 mm, respectively. All these results illustrate how difficult is to attribute a biological response to a given molecular descriptor. Interestingly, Figure 1 shows that phenylaminojuglones displaying high HGI (i.e., 7, 13) have the highest prooxidant activities as shown by the TBARS production, suggesting a potential link between oxidative stress and antibacterial activity. Supporting the role of oxidative stress during chronic gastritis associated with *H. pylori* infection, it should be noted that the administration of coenzyme q10 decreases mucosal inflammation in such patients [56].

In conclusion, compound 7 is a promissory anti-*pylori* compound already active as soon as 30 min of incubation at a very low concentration (0.56 *μ*M). When using at 4.5 *μ*M (1.6 *μ*g/mL), the calculated halo of growth inhibition for 7 was 32.25 mm. These preliminary results make 7 an interesting lead molecule modulated by other substituting groups and to conduct further assays.

## Figures and Tables

**Table 1:** Effect of juglone and their arylamino analogs on *Helicobacter pylori* growth.

Dose (*μ*g)	0.0	0.1	0.2	0.4	0.8	1.6
Compound number	Halo of growth inhibition (mm)					
2	0.00	9.25	10.50	11.50	13.25	14.50
4	0.00	8.50	9.75	9.75	11.75	13.25
5	0.00	7.50	8.25	10.00	10.50	11.00
6	0.00	9.50	10.50	11.25	12.50	13.50
7	0.00	20.00	22.50	24.50	27.25	32.25
8	0.00	8.75	10.00	10.50	11.75	13.75
9	0.00	7.50	8.75	9.75	10.25	10.75
10	0.00	10.00	11.25	14.50	16.50	23.50
11	0.00	17.75	21.25	24.75	26.00	27.75
12	0.00	8.25	8.75	9.75	10.50	12.75
13	0.00	19.75	21.50	23.75	25.00	28.50
14	0.00	17.75	19.50	20.00	20.50	22.25
15	0.00	9.50	10.75	11.00	11.75	12.75
Metronidazole^∗^	0.00	—	7.83	8.67	9.33	11.67

Values are expressed as HGI (in mm) and they are means of two separated experiments. ^∗^Reference drug.

## Abbreviations

CFU: Colony-forming units
HGI: Halo of growth inhibition
MDA: Malondialdehyde
TSA: Trypticase soy agar
TSB: Trypticase soy broth.

## Supplementary Materials

Supplementary Materials Description of the general procedure for the preparation of phenylaminojuglone 4–7 and 8–15 and spectral data of nuclear magnetic resonance (^1^H NMR and ^13^C NMR) and high-resolution mass spectrometry (HRMS).
