# Supplementary material for: In Vitro Inhibition of Helicobacter pylori Growth by Redox Cycling Phenylaminojuglones
Source: Oxid Med Cell Longev. 2018 Apr 24;2018:1618051. doi: 10.1155/2018/1618051 (PMC5941820; doi:10.1155/2018/1618051)
Supplement: Supplementary Materials — Description of the general procedure for the preparation of phenylaminojuglone 4–7 and 8–15 and spectral data of nuclear magnetic resonance (1H NMR and 13C NMR) and high-resolution mass spectrometry (HRMS). [file 1618051.f1.pdf]

Supplementary Data:

*General Procedure for the preparation of phenylaminojuglone **4-7** and **8-15**.*

A suspension of 1,5-dihydroxynaphthalene (**1**; 1.25 mmol), rose bengal (20 mg; 0.02 mmol) as sensitizer and water (150 mL), in a round bottom flask, was exposed to green LEDs for 5 h while a gentle stream of air is bubbled through the solution. Then is added the respective phenylamine (1.5 mmol), and the solution was stirred at rt for 4 h. Work up followed by column chromatography over silica gel (3:1 petroleum ether/ethyl acetate) yield pure compounds **4-7** and **8**-1**5**.

2-(phenylamino)-5-hydroxynaphthalene-1,4-dione **4** *3-(phenylamino)-5-hydroxynaphthalene-1,4-dione* **8**

Prepared from 1,5-dihydroxynaphthalene **1** and phenylamine. Compound **4**, dark brown solid, mp, 149-151°C. IR (KBr) ν_{max} cm^{-1}: 3450 (OH), 3278 (NH), 1584 (C=O). ^{1}H-NMR (400 MHz, $CDCl_3$) δ: 6.38 (s, 1H, H-3), 7.22 (m, 4H, H-2' + H-4' + H-6' + H-6), 7.43 (dd, 2H, *J* = 7.9, 7.8 Hz, H-3' + H-8'), 7.53 (bs, 1H, -NH), 7.64 (m, 2H, H-7 + H-8), 11.57 (s, 1H, OH). ^{13}C-NMR (100 MHz, $CDCl_3$): δ 103.92, 114.58, 118.77, 122.63, 122.78, 125.85, 129.74, 132.23, 133.16, 134.73, 137.16, 137.84, 144.65, 161.73, 183.12, 186.15. HRMS (APCI): $[M+H]^+$ calculated for $C_{16}H_{11}NO_3$: 266.08172 found 266.08061.

Compound **8**, light brown solid, mp, 221-223°C. IR (KBr) ν_{max} cm^{-1}: 3440 (OH), 3278 (NH), 1626 (C=O). ^{1}H-NMR (400 MHz, $CDCl_3$) δ: 5.86 (s, 1H, H-2), 7.26 (m, 6H, H-3' + H-4' + H-5' + H-6' + H-6), 7.46 (bs, 1H, -NH), 7.52 (dd, 1H, *J* = 8.0, 7.8 Hz, H-7), 7.67 (d, 1H, *J* = 7.4 Hz, H-8), 12.89 (s, 1H, OH). ^{13}C-NMR (100 MHz, $CDCl_3$): δ 17.71, 102.09, 114.59, 119.25, 124.86, 126.09, 127.20, 127.24, 130.42, 131.48, 133.26, 134.21, 135.05, 146.54, 161.06, 181.40, 189.75. HRMS (APCI): $[M+H]^+$ calculated for $C_{16}H_{11}NO_3$: 266.08172 found 266.08062.

2-((2-methylphenyl)amino)-5-hydroxynaphthalene-1,4-dione **5** *and 3-((2-methylphenyl)amino)- 5-hydroxynaphthalene-1,4-dione* **9**

Prepared from 1,5-dihydroxynaphthalene **1** and 2-methylphenylamine. Compound **5**, orange solid, mp. 210-212°C. IR (KBr) ν_{max} cm^{-1}: 3449 (OH), 3301 (NH), 1594 (C=O). ^{1}H-NMR (400 MHz, $CDCl_3$) δ: 2.30 (s, 3H, Me), 5.93 (s, 1H, H-3), 7.19 (dd, 1H, *J* = 7.4, 2.1 Hz, H-6), 7.22 (m, 1H, H-4' or H-6'), 7.29 (m, 3H, -NH + H-5' + H-6' or H-4'), 7.30 (dd, 1H, *J* = 7.0, 6.6 Hz, H-3'), 7.64 (m, 2H, H-7 + H-8), 11.59 (s, 1H, OH). ^{13}C-NMR (100 MHz, $CDCl_3$): δ 17.73, 103.70, 114.09, 118.79, 122.54, 124.87, 127.06, 127.21, 131.44, 133.21, 133.26, 135.18, 137.81, 145.66, 161.74, 182.95, 186.26. HRMS (APCI): $[M+H]^+$ calculated for $C_{17}H_{13}NO_3$: 280.09737 found 280.09628.

Compound **9**, dark brown solid, mp, 150-152°C. IR (KBr) ν_{max} cm^{-1}: 3449 (OH), 3314 (NH), 1626 (C=O). ^{1}H-NMR (400 MHz, $CDCl_3$) δ: 2.29 (s, 3H, Me), 5.86 (s, 1H, H-2), 7.26 (m, 6H, H-3' + H-4' + H-5' + H-6' + H-6), 7.46 (bs, 1H, -NH), 7.52 (dd, 1H, *J* = 8.0, 7.8 Hz, H-7), 7.67 (d, 1H, *J* = 7.4

Hz, H-8), 12.89 (s, 1H, OH). ^{13}C-NMR (100 MHz, $CDCl_3$): δ 17.71, 102.09, 114.59, 119.25, 124.86, 126.09, 127.20, 127.24, 130.42, 131.48, 133.26, 134.21, 135.05, 146.54, 161.06, 181.40, 189.75. HRMS (APCI): $[M+H]^+$ calculated for $C_{17}H_{13}NO_3$: 280.09737 found 280.09625.

2-((4-methoxyphenyl)amino)-5-hydroxynaphthalene-1,4-dione **6** *and 3-((4-methoxyphenyl)amino)- 5-hydroxynaphthalene-1,4-dione* **12**.

Prepared from 1,5-dihydroxynaphthalene **1** and 4-methoxyphenylamine. Compound **6**, dark brown solid, mp, 212-214°C. IR (KBr) ν_{max} cm^{-1}: 3440 (O-H), 3285 (N-H), 1645 (C=O), 1623 (C=O). ^{1}H-NMR (300 MHz, $CDCl_3$): δ 3.84 (s, 3H, OMe), 6.11 (s, 1H, H-3), 6.96 (d, 2H, *J* = 7.9 Hz, H-3' + H-5'), 7.20 (m, 3H, H-6 + H-2' + H-6'), 7.51 (t, 1H, *J* = 7.6 Hz, 7-H), 7.64 (m, 2H, H-8 + NH), 12.95 (s, 1H, OH). ^{13}C-NMR (75 MHz, $CDCl_3$): δ 55.71, 101.57, 115.09 (2C), 119.43, 125.07 (2C), 126.22, 129.69, 130.47, 134.30, 137.94, 146.48, 158.05, 161.12, 181.56, 189.84. HRMS (APCI) calculated for $C_{17}H_{13}NO_4$: 295.08446 $[M+H]^+$; found 295.09110.

Compound **12**, dark brown solid, mp, 205-207°C; IR (KBr) ν_{max} cm^{-1}: 3448 (O-H), 3274 (N-H), 1640 (C=O), 1625 (C=O). ^{1}H-NMR (400 MHz, DMSO-d_6): δ 3.78 (s, 3H, OMe), 5.86 (s, 1H, H-2), 7.02 (d, 2H, *J* = 8.8 Hz, H-3' + H-5'), 7.24 (d, 1H, *J* = 8.4 Hz, H-6), 7.29 (d, 2H, *J* = 8.8 Hz, H-2' + H-6'), 7.45 (d, 1H, *J* = 6.9 Hz, H-8), 7.73 (dd, 2H, *J* = 7.8, 8.1 Hz, H-7), 9.19 (s, 1H, NH), 11.55 (s, 1H, OH). ^{13}C-NMR (100 MHz, DMSO-d_6): δ 55.70, 101.66, 114.66, 114.94 (2C), 117.95, 122.46, 126.13 (2C), 130.82, 133.48, 137.94, 147.31, 157.44, 160.87, 181.93, 186.06. HRMS (APCI) calculated for $C_{17}H_{13}NO_4$: 295.08446 $[M+H]^+$; found 295.09102.

2-((3,4,5-trimethoxyphenyl)amino)-5-hydroxynaphthalene-1,4-dione **7** *and 3-((3,4,5-trimethoxyphenyl)amino)- 5-hydroxynaphthalene-1,4-dione* **13**.

Prepared from 1,5-dihydroxynaphthalene **1** and 3,4,5-trimethoxyphenylamine. Compound **7**, dark red solid, mp, 193-195°C; IR (KBr) ν_{max} cm^{-1}: 3449 (O-H), 3298 (N-H), 1682 (C=O), 1630 (C=O). ^{1}H-NMR (400 MHz, DMSO-d_6): δ 3.67 (s, 3H, OMe), 3.77 (s, 6H, OMe), 6.05 (s, 1H, H-3), 6.70 (s, 2H, H-2' + H-6'), 7.24 (d, 1H, *J* = 8.4 Hz, H-6), 7.45 (d, 1H, *J* = 7.3 Hz, H-8), 7.73 (dd, 2H, *J* = 7.6, 8.3 Hz, H-7), 9.11 (s, 1H, NH), 11.51 (s, 1H, OH). ^{13}C-NMR (100 MHz, DMSO-d_6): δ 56.07, 60.18 (2C), 101.92 (2C), 102.58, 114.23, 117.65, 122.25, 133.02, 133.69, 135.28, 137.62, 146.31, 153.23 (2C), 160.50, 181.84, 185.52. HRMS (APCI) calculated for $C_{19}H_{17}NO6$: 355.10559 $[M+H]^+$; found 355.11198.

Compound **13**, dark purple solid, mp 188-190°C; IR (KBr) ν_{max} cm^{-1}: 3448 (O-H), 3369 (N-H), 1735 (C=O), 1629 (C=O). ^{1}H-NMR (400 MHz, DMSO-d_6): δ 3.68 (s, 3H, OMe), 3.78 (s, 6H, OMe), 6.06 (s, 1H, H-3), 6.70 (s, 2H, H-2' + H-6'), 7.25 (d, 1H, *J* = 8.4 Hz, H-6), 7.46 (d, 1H, *J* = 7.3 Hz, H-8), 7.73 (dd, 2H, *J* = 7.7, 8.2 Hz, H-7), 9.12 (s, 1H, NH), 11.52 (s, 1H, OH). ^{13}C-NMR (100 MHz, DMSO-d_6): δ 56.41, 60.53 (2C), 102.26 (2C), 102.92, 114.57, 118.00, 122.60, 133.37, 134.04, 135.63, 137.97, 146.67, 153.57 (2C), 160.84, 182.19, 185.87. HRMS (APCI) calculated for $C_{19}H_{17}NO6$: 355.10559 $[M+H]^+$; found 355.11206.

3-((4-hydroxyphenyl)amino)-5-hydroxynaphthalene-1,4-dione **10** was prepared from 1,5-dihydroxynaphthalene **1** and 4-hydroxyphenylamine. Compound **10**, brown solid, mp, 243-245°C; IR (KBr) ν_{max} cm^{-1}: 3383 (O-H), 3339 (O-H), 3286 (N-H), 1718 (C=O), 1630 (C=O). ^{1}H-NMR (400 MHz, DMSO-d_6): δ 5.82 (s, 1H, H-3), 6.85 (d, 2H, *J* = 8.5 Hz, H-3' + H-5'), 7.16 (d, 2H, *J* = 8.5 Hz, H-2' + H-6'), 7.23 (d, 1H, *J* = 8.4 Hz, H-6), 7.45 (d, 1H, *J* = 7.3 Hz, H-8), 7.72 (dd, 2H, *J* = 7.8, 8.1 Hz, H-7), 9.0 (s, 1H, NH), 9.54 (s, 1H, OH-4'), 11.47 (s, 1H, OH). ^{13}C-NMR (100 MHz, DMSO-d_6): δ 101.54, 114.67, 116.26 (2C), 117.94, 122.36, 126.25 (2C), 129.25, 133.60, 137.91, 147.45, 155.84, 160.90, 181.80, 186.18. HRMS (APCI) calculated for $C_{16}H_{11}NO_4$: 281.06881 [M+H]$^+$; found 281.07541.

3-((3-methoxyphenyl)amino)- 5-hydroxynaphthalene-1,4-dione **11**.

Prepared from 1,5-dihydroxynaphthalene **1** and 3-methoxyphenylamine. Compound **11**, dark purple solid, mp 179-181°C; IR (KBr) ν_{max} cm^{-1}: 3448 (O-H), 3330 (N-H), 1640 (C=O), 1624 (C=O). ^{1}H-NMR (400 MHz, DMSO-d_6): δ 3.78 (s, 3H, OMe), 5.85 (s, 1H, H-2), 7.01 (d, 2H, *J* = 8.9 Hz, H-3' + H-5'), 7.24 (d, 1H, *J* = 8.4 Hz, H-6), 7.28 (d, 2H, *J* = 8.8 Hz, H-2' + H-6'), 7.44 (d, 1H, *J* = 7.4 Hz, H-8), 7.72 (dd, 2H, *J* = 7.6, 8.3 Hz, H-7), 9.18 (s, 1H, NH), 11.54 (s, 1H, OH). ^{13}C-NMR (100 MHz, DMSO-d_6): δ 55.70, 101.66, 114.66, 114.94 (2C), 117.95, 122.46, 126.13 (2C), 130.82, 133.48, 137.94, 147.31, 157.44, 160.87, 181.93, 186.07. HRMS (APCI) calculated for $C_{17}H_{13}NO_4$: 295.08446 [M+H]$^+$; found 295.09109.

3-((4-((4-Aminophenyl)sulfonyl)phenyl)amino)-5-hydroxynaphthalene-1,4-dione **14**.

Prepared from 1,5-dihydroxynaphthalene **1** and dapsone. Compound **14**, orange solid, mp, 300-302°C; IR (KBr) ν_{max} cm^{-1}: 3462 (O-H), 3373 (N-H), 1635 (C=O), 1614 (C=O). ^{1}H-NMR (400 MHz, DMSO-d_6): δ 6.18 (s, 2H, NH_2), 6.29 (s, 1H, H-2), 6.64 (d, 2H, *J* = 8.7 Hz, H-3'' + H-5''), 7.27 (d, 1H, *J* = 8.3 Hz, H-6), 7.46 (dd, 1H, *J* = 7.9, 9.5 Hz, H-8), 7.57 (m, 4H, H-3' + H-5' + H-2'' + H-6''), 7.73 (dd, 2H, *J* = 7.9, 7.9 Hz, H-7), 7.86 (d, 2H, *J* = 8.6 Hz H-2' + H-6'), 9.45 (s, 1H, NH), 11.50 (s, 1H, OH). ^{13}C-NMR (100 MHz, DMSO-d_6): δ 105.03, 113.43 (2C), 118.08, 122.93 123.24 (2C), 125.98, 128.30 (2C), 128.88, 129.72 (2C), 133.00, 137.90, 138.80, 142.58, 145.28, 153.95, 160.83, 182.76, 185.51. HRMS (APCI) calculated for $C_{22}H_{16}N_2O_5S$: 420.07799 [M+H]$^+$; found 420.08393.

3-((4'-Amino-[1,1'-biphenyl]-4-yl)amino)-5-hydroxynaphthalene-1,4-dione **15.**

Prepared from 1,5-dihydroxynaphthalene **1** and benzidine. Compound **15**, dark purple solid, mp, 268-270°C. IR (KBr) ν_{max} cm^{-1}: 3446 (O-H), 3359 (N-H), 3280 (NH_2), 1685 (C=O), 1623 (C=O). ^{1}H-NMR (300 MHz, DMSO-d_6): δ 5.25 (s, 2H, NH_2), 6.07 (s, 1H, H-2), 6.65 (d, 2H, *J* = 8.2 Hz, H-benci), H- 7.24 (d, 1H, *J* = 8.4 Hz, H-benci), 7.37 (t, 4H, *J* = 8.1 Hz, H-benci), 7.45 (d, 1H, *J* = 7.3 Hz, H-6), 7.60 (d, 2H, *J* = 8.3 Hz, H-8 + H-benci), 7.72 (t, 2H, *J* = 7.9 Hz, H-7), 9.29 (s, 1H, NH),

11.56 (s, 1H, OH). ^{13}C-NMR (75 MHz, DMSO-d_6): δ 102.23, 114.51, 114.56 (2C), 117.92, 122.52, 124.32 (2C), 126.23 (2C), 126.81, 127.31 (2C), 133.23, 135.74, 137.90, 138.00, 146.35, 148.66, 160.76, 182.15, 185.81. HRMS (APCI) calculated for $C_{22}H_{16}N_2O_3$: 356.11609 $[M+H]^+$; found 356.11230.
